# Association between dietary saturated fat with cardiovascular disease risk markers and body composition in healthy adults: findings from the cross-sectional BODYCON study

**DOI:** 10.1186/s12986-022-00650-y

**Published:** 2022-03-03

**Authors:** Ezgi Ozen, Rada Mihaylova, Michelle Weech, Sam Kinsella, Julie A. Lovegrove, Kim G. Jackson

**Affiliations:** Hugh Sinclair Unit of Human Nutrition, Department of Food and Nutritional Sciences and Institute for Cardiovascular and Metabolic Research and Institute for Food, Nutrition and Health, University of Reading, Harry Nursten Building, Whiteknights, Pepper Lane, Reading, RG6 6DZ UK

**Keywords:** Body composition, Abdominal obesity, Dietary fat quality, SFA intake, DXA

## Abstract

**Background:**

Diets high in saturated fatty acids (SFAs) and greater abdominal obesity are both associated with raised low-density lipoprotein cholesterol (LDL-C) concentrations, an independent cardiovascular disease (CVD) risk marker. Although reducing SFA intake is a public health strategy for CVD prevention, the role of body fat distribution on the relationship between SFA and LDL-C is unclear. Therefore, our objective was to investigate whether the association between dietary SFAs and LDL-C concentrations is related to body composition.

**Methods:**

In the BODYCON (impact of physiological and lifestyle factors on body composition) study, 409 adults [mean age 42 ± 16 years and median BMI of 23.5 (21.5–25.9) kg/m^2^] underwent a measure of body composition by dual energy x-ray absorptiometry, assessment of habitual dietary intake using a 4-day weighed food diary and physical activity level using a tri-axial accelerometer. Blood pressure was measured, and a fasting blood sample was collected to determine cardiometabolic disease risk markers. Correlations between body composition, circulating risk markers and dietary macronutrients were assessed prior to multivariate regression analysis. The effect of increasing intakes of dietary SFA on outcome measures was assessed using ANCOVA after adjusting for covariates.

**Results:**

Abdominal visceral adipose tissue (VAT) mass was moderately positively correlated with total cholesterol (TC), LDL-C, systolic blood pressure (SBP), diastolic blood pressure and HOMA-IR (r_s_ = 0.25–0.44, *p* < 0.01). In multiple regression analysis, 18.3% of the variability in LDL-C was explained by SFA intake [% total energy (TE)], abdominal VAT mass, carbohydrate%TE and fat%TE intakes. When data were stratified according to increasing SFA%TE intakes, fasting TC, LDL-C and non-high-density lipoprotein-cholesterol were higher in Q4 compared with Q2 (*p* ≤ 0.03). SBP was higher in Q4 versus Q3 (*p* = 0.01). Android lean mass was also higher in Q3 versus Q1 (*p* = 0.02). Other anthropometric and CVD risk markers were not different across quartile groups.

**Conclusions:**

Although dietary SFA was found to explain 9% of the variability in LDL-C, stratification of data according to quartiles of SFA intake did not reveal a dose-dependent relationship with LDL-C concentration. Furthermore**,** this association appeared to be independent of abdominal obesity in this cohort.

*Clinical Trail registration: Trial registration*: clinicaltrials.gov as NCT02658539. Registered 20 January 2016, https://clinicaltrials.gov/ct2/show/NCT02658539.

**Supplementary Information:**

The online version contains supplementary material available at 10.1186/s12986-022-00650-y.

## Introduction

Diet is one of the most important modifiable risk factors for cardiovascular diseases (CVDs), with studies reporting a link between high intakes of dietary saturated fatty acids (SFAs) and elevated low-density lipoprotein-cholesterol (LDL-C), a well-documented independent risk factor for this disease [1, 2]. Although many studies have investigated the effect of reducing dietary SFA intake on the fasting lipid profile, replacement with unsaturated fatty acids was found to be more beneficial compared to carbohydrates or protein [3, 4]. Thus, current UK recommendations for CVD prevention are to decrease dietary SFA intake to less than 10% of total energy (TE) via replacement with polyunsaturated (PUFAs) and monounsaturated fatty acids (MUFAs) [5]. However, there is also consistent evidence suggesting no beneficial effect of reducing dietary SFA intake on CVD mortality [6–8]. These discrepancies between studies indicate that there may be other factors affecting this relationship.

Obesity is a rapidly growing global public health problem affecting over one third of the world’s population [9, 10]. An excessive accumulation of body fat is positively associated with the risk of cardiometabolic diseases such as CVD and type 2 diabetes [11]. Body mass index (BMI) has been used routinely at a population level to assess adiposity and identify people with increased metabolic disease risk. However, body fat distribution is now considered to be a better indicator of chronic disease risk than BMI, with fat accumulation in the abdominal area [especially visceral adipose tissue (VAT)] associated with greater CVD risk compared with gynoid adiposity [12–15]. Moderately elevated LDL-C concentrations and insulin resistance have been observed in people with increased abdominal fat accumulation [16–18]. As a result, there is a considerable interest in the physiological and lifestyle characteristics that influence body fat distribution [19, 20].

Storage of body fat is influenced by non-modifiable factors such as age and sex [21], but also by modifiable lifestyle factors such as diet [22]. Studies have investigated the effect of dietary fat quality on body composition, with differential associations shown between dietary SFA (positive) and PUFA/MUFA (negative) with abdominal obesity [23–25]. Although the impact of dietary SFAs on LDL-C concentrations has been shown in many studies, the effect of body composition on this relationship is poorly understood. A small number of studies have reported BMI to be inversely associated with the LDL-C response to reduced SFA intake [26]. As dietary SFAs are reported to influence both LDL-C concentrations and body composition, the effect of dietary SFAs on LDL-C, therefore, might be related to its effect on body fat content and distribution.

Thus, the purpose of this study was to investigate whether the impact of dietary SFA on LDL-C was associated with body composition. We hypothesized that higher SFA intakes are related to increased LDL-C concentrations due to greater fat accumulation in the abdominal area.

## Methods

### Subjects

Healthy men and women (n = 409) aged 18–70 years were recruited from Reading and the surrounding area (UK), from 2014 through 2019 using posters, pamphlets and by contacting previous volunteers registered on the Hugh Sinclair Unit of Human Nutrition volunteer database at the University of Reading. A Medical and Lifestyle questionnaire was used to assess the suitability of interested volunteers before potentially eligible individuals were invited to attend a screening session in which they were provided with detailed information about the study before signing a consent form. All subjects were assessed after fasting overnight for 12 h. During the screening visit, blood pressure and anthropometric measurements were taken and a fasting blood sample was collected for the measurement of fasting blood lipids [total cholesterol (TC), triacylglycerol (TAG) and high density lipoprotein cholesterol (HDL-C)], glucose, kidney and liver function markers (alkaline phosphatase, alanine aminotransferase, γ-glutamyl transferase, serum creatinine, total bilirubin and uric acid) by using the ILAB 600 clinical chemistry analyser (Werfen Ltd, Warrington,UK). To determine the haemoglobin level, a further blood sample was sent to the Royal Berkshire Hospital Pathology Department (Reading, UK). All participants whose screening measurements matched the following inclusion criteria were invited to participate in the study: BMI 18.5–39.9 kg/m^2^, TC < 7.8 mmol/l, TAG < 2.3 mmol/l, fasting blood glucose < 7.8 mmol/l, haemoglobin > 115 g/l for women and 130 g/l for men. Exclusion criteria included the following: having suffered a myocardial infarction/stroke in the past 12 months, history of diabetes or other endocrine disorders, bowel disease, cholestatic liver disease, pancreatitis, cancer, being on medication for hyperlipidemia, hypertension, inflammation or hypercoagulation, being on a weight reducing diet and excessive alcohol consumption (< 14 units/wk). Furthermore, due to the use of the dual energy x ray absorptiometry (DXA) to assess body composition, further exclusion criteria included arthritis or fracture deformity of spine or femur, history of bone related surgeries, radio-opaque implants or implanted medical devices. Females were also excluded if they were breast feeding, may be pregnant or planning a pregnancy in the next 12 months.

### Study design

Impact of physiological and lifestyle factors on body composition (BODYCON) was a single-centered observational cross-sectional study conducted in the Hugh Sinclair Unit of Human Nutrition at the University of Reading. The NHS and University of Reading Research Ethics Committees (reference numbers 14/SC/1095 and 13/55, respectively) both gave a favorable ethical opinion for conduct. This study was carried out in accordance with the Declaration of Helsinki and was registered at www.clinicaltrials.gov (NCT02658539).

Participants attended a single study visit. For the day prior to this visit, participants were requested to abstain from strenuous exercise and consuming alcohol. A low-fat evening study meal and low-nitrate water (Buxton mineral water, Nestlé waters, UK) were provided by the researchers and participants were asked not to consume anything apart from this water after their evening meal. Before starting the study visit, a spot urine sample was collected and urine osmolarity was measured using an Osmocheck device (Vitech Scientific Ltd., UK) to ensure participants were sufficiently hydrated for the body composition measurements and asked to complete a pre-DXA scan questionnaire. Weight, waist and hip circumferences were measured, followed by clinic blood pressure. Total body composition was assessed by DXA scan before a fasting blood sample was taken to measure cardiometabolic disease risk markers. Additionally, in the few days before their visit participants were asked to complete a 4-day weighed food diary for 3 consecutive weekdays and 1 weekend day while wearing a triaxial Actigraph activity monitor (ActiGraph, Florida, US) during the same time to assess dietary intake and physical activity levels, respectively. Premenopausal women not taking oral contraceptives attended their main study visit during the same phase of their menstrual cycle (days 1–7).

### Anthropometric and blood pressure measurements

Anthropometric and body composition measurements were performed with participants wearing light clothing and no shoes or metal objects. Height was measured to the nearest 1 cm using a stadiometer, facing forwards, and standing as straight as possible with their arms hanging loosely by their side and their head in the Frankfort plane. Body weight and BMI were determined by using a bioelectrical impedance analyser (Tanita BC-418, TANITA UK Ltd, Middlesex, UK) and 1 kg was automatically deducted to account for the weight of the subject’s light clothing. Waist circumference (WC) was measured at the midpoint between the lowest ribs and the top of the iliac crest while hip circumference was measured at the largest circumference around the buttocks. Both measurements were taken by a trained researcher while participants were standing straight after a gentle expiration. A non-stretch tape measure (Seca, UK) was used for both measures. The waist to hip ratio (WHR) and waist to height ratio (WHtR) were calculated as estimates of body fat distribution.

Blood pressure was measured three times using an Omron blood pressure monitor (Omron M3 digital automatic upper arm blood pressure monitor, Omron Healthcare Co UK Ltd.) and the average systolic blood pressure (SBP) and diastolic blood pressure (DBP) were calculated. Pulse pressure was determined by subtracting DBP from SBP.

### Visceral adiposity, fat mass and lean mas index calculations

Anthropometric indices were calculated to determine their relationship with dietary SFA and cardiometabolic disease risk markers. These included the visceral adiposity index (VAI = waist circumference/(39.68 + (1.88 × BMI))  × (TAG(mmol/L)/1.03) × (1.31/HDL-C(mmol/L)) for men and VAI = waist circumference/(36.58 + (1.89 × BMI)) × (TAG(mmol/L)/0.81) × (1.52/HDL-C(mmol/L)) for women as an indicator of visceral adipose tissue function [27]), fat mass index (FMI = fat mass(kg)/height in m^2^) and lean mass index (LMI = lean mass(kg)/height in m^2^) [28].

### Assessment of dietary intake

Habitual dietary intake was evaluated by using a 4-day weighed diet diary. To increase accuracy, an electronic kitchen scale and a selection of food portion sizes from the Food Atlas to record meals consumed outside of home [29] were provided to the participants. Instructions on how to complete the diary were given both verbally and in written form by the researchers. For each subject, nutrient and energy intakes were calculated using Dietplan 7 (Forestfield Software) and the total dietary intakes were divided by the number of days recorded to give mean daily intakes. Data entered on Dietplan was checked by a single researcher at the end of the study. For dietary data inclusion, participants were required to complete at least 3 days of the diet diary and report feasible dietary intakes between 500 and 3500 kcal per day for women and 800 and 4000 kcal per day for men. Individuals with dietary intakes outside of these ranges have been previously reported to be under and over reporters [30].

### Physical activity

A tri-axial accelerometer was used to measure physical activity levels (Actigraph wGT3X+, Actigraph, LLC). Participants were asked to wear the accelerometer for 4 consecutive days including 3 weekdays and 1 weekend day and keep an activity diary for data cleaning purposes. It was worn around the abdomen above their right hip bone, and they were asked to remove the device only for showering or during swimming. Device initialization, data processing and analysis were conducted using Actilife Data Analysis Software (Version 6.11.5) as previously described [31]. Raw data was collected at a 30 Hz sample rate. For inclusion in the physical activity analysis, participants were required to have produced counts on their activity monitor for ≥ 3-days (> 600 min/day of wear time) [32]. Non-wear-time was defined as ≥ 60 min of zero activity counts [33]. Data were summarized in 60-s epochs and cut-points were used to classify wear time as: sedentary behaviour (< 100 counts/min), light/lifestyle physical activity (760–1951 counts/min), moderate physical activity (1952–5724 counts/min) and vigorous physical activity (≥ 5725 counts/min) [34]. For the purposes of the data analysis, the time spent in moderate and vigorous physical activity was combined. Mean energy expenditure from physical activity (EE_PA_) was calculated as kcal/day.

### Details of the DXA procedure

Prior to the DXA scan assessment, participants changed into clothing without zips and metal buttons or a disposable hospital garment and all metal artefacts were removed. Whole body composition was measured by Lunar iDXA (GE Healthcare, UK) and two operators performed the scanning and followed the manufacturer’s guidelines for volunteer positioning and for scan acquisition. Participants laid supine on the Lunar iDXA scanning table with knees and ankles positioned together using the Lunar Velcro supports. Arms were positioned to the side of the body, with palms facing towards the body and participants were required to lie still during the total body composition scan. All scans were analysed using enCORE Software, version 15 (GE Healthcare, UK) with the advance software package CoreScan, which also estimates the mass and volume of visceral fat within the abdomen. The machine’s performance was checked daily by running a quality assurance test according to the manufacturer’s instructions before each scanning session.

### Biochemical analysis

Blood samples collected into the serum separator and K_3_EDTA blood tubes were centrifuged at 1700 × *g* (3000 rpm) for 15 min at room temperature and 4 °C, respectively before aliquoting into Eppendorf tubes and stored at − 20 °C. Fasting serum lipids (non-esterified fatty acids (NEFA) (Alpha Laboratories Ltd., Hampshire, UK), TC, HDL-C and TAG), glucose, C-reactive protein (CRP), and ɣ-glutamyl transferase (GGT) were quantified in the main study visit sample by using the ILAB 600 clinical chemistry analyser with reagents from Werfen (Werfen (UK) Ltd., Warrington, UK). Plasma uric acid was measured using RX Daytona Plus clinical chemistry analyser (Randox Laboratories Ltd., County Antrim, UK) using a kit supplied by Randox. The Friedewald formula was used to estimate fasting LDL-C concentrations [35]. Non-HDL-C was calculated by subtracting HDL-C from TC. ELISA kits were used to analyse serum insulin (Dako Ltd., High Wycombe, UK) and plasma adiponectin (Quantikine kit, R&D Systems, Europe Ltd.) concentrations. Homeostatic model assessment for insulin resistance (HOMA-IR) was calculated by using the following equation: [fasting insulin (pmol/l) × fasting glucose (mmol/l)]/135 [36]. Serum 25 hydroxyvitamin D_2_ and 25 hydroxyvitamin D_3_ was measured by the LGC group (LGC Ltd., Middlesex, UK) and summed to obtain total 25 hydroxy vitamin D (25(OH)D).

### Statistical analysis

Statistical analyses were performed using IBM SPSS Statistics version 25 (SPSS Inc., IL, US). Data was presented as mean ± standard deviation (SD) for normally distributed variables and as median (interquartile range) for non-normally distributed variables in Tables 1 and 2. Normality was assessed using the Kolmogorov–Smirnov test and Q–Q plots. The logarithms or square root transformations were used for several outcome measures including BMI, body fat mass, abdominal VAT mass, dietary protein and trans-fat, TAG, LDL-C: HDL-C ratio, TC: HDL-C ratio, NEFA, CRP, GGT, adiponectin, insulin and HOMA-IR, steps/day, EE_PA_, and percentage time spent performing moderate to vigorous physical activity. Parametric independent sample t tests were used for normally distributed and transformed data to determine the differences between the male and female groups. Spearman’s correlations were used to analyse relationships between cardiometabolic disease risk markers with body composition measurements and dietary macronutrients in the whole group and in men and women separately (Spearman’s Rho (r_s_) = 0–0.3 considered a weak correlation, r_s_ = 0.3–0.7 moderate and r_s_ = 0.7–1.0 strong). Stepwise multiple linear regression analysis was performed using P-in of 0.05 and P-out of 0.01 to establish the independent associations between LDL-C and abdominal VAT mass with the anthropometric measures, cardiometabolic disease risk markers and dietary macronutrients.

**Table 1:** Characteristics of BODYCON study participants

	All (n = 409)	Men (n = 190)	Women (n = 219)	*p* value^a^
*Outcome measures*^b^				
Age, years	42 ± 16	42 ± 15	42 ± 16	0.93
Weight, kg	70.4 ± 14.0	78.3 ± 12.2	63.5 ± 11.7	< 0.01
Height, m	1.71 ± 0.01	1.78 ± 0.07	1.64 ± 0.07	< 0.01
BMI, kg/m^2^	23.5 (21.5–23.9)	24.2 (22.7–26.5)	22.5 (20.8–25.4)	0.01
WC, cm	83.8 ± 11.9	89.1 ± 10.3	79.2 ± 11.2	< 0.01
HC, cm	101 ± 9	102 ± 9	100 ± 10	0.04
WHR	0.83 ± 0.08	0.88 ± 0.07	0.79 ± 0.08	< 0.01
WHtR	0.49 ± 0.07	0.50 ± 0.06	0.48 ± 0.07	< 0.01
*Blood pressure*, mmHg				
Systolic	120 ± 14	124 ± 11	117 ± 15	< 0.01
Diastolic	72 ± 9	74 ± 9	70 ± 9	< 0.01
Pulse pressure	48 ± 11	50 ± 10	47 ± 10	< 0.01
*Body composition measures*				
Body fat, %	28.3 ± 8.4	23.7 ± 7.2	32.3 ± 7.4	< 0.01
Fat mass, kg	19.0 (14.3–25.0)	17.8 (12.9–24.9)	19.3 (15.5–25.2)	0.01
Lean mass, kg	48.4 ± 10.5	40.7 ± 5.7	57.2 ± 7.4	< 0.01
Trunk fat mass, kg	10.4 ± 5.0	10.9 ± 5.2	10.0 ± 4.9	0.09
Abdominal VAT, g	393 (178–811)	691 (367–1240)	237 (99–440)	< 0.01
Android fat, %	30.5 ± 12.1	29.1 ± 12.1	31.8 ± 11.8	0.03
Gynoid fat, %	32.2 ± 9.9	24.9 ± 7.0	38.7 ± 7.2	0.01
A/G fat ratio	0.96 ± 0.29	1.13 ± 0.28	0.80 ± 0.21	0.01
*Body Composition Indexes*				
FMI, kg/m^2^	7.05 ± 2.93	6.10 ± 2.43	7.88 ± 3.09	0.01
LMI, kg/m^2^	16.4 ± 2.2	18.0 ± 1.7	15.0 ± 1.5	0.01
VAI	1.01 ± 0.68	1.03 ± 0.71	1.00 ± 0.65	0.66
*Biochemistry*				
TC, mmol/L	5.13 ± 1.10	5.05 ± 1.18	5.20 ± 1.02	0.10
TAG, mmol/L	0.83 (0.66–1.16)	0.93 (0.69–1.39)	0.79 (0.64–1.02)	0.01
HDL-C, mmol/L	1.65 ± 0.40	1.51 ± 0.40	1.78 ± 0.36	0.01
LDL-C, mmol/L	3.03 ± 0.93	3.07 ± 1.00	2.99 ± 0.86	0.66
Non-HDL-C, mmol/L	3.48 ± 1.00	3.55 ± 1.07	3.43 ± 0.94	0.34
TC:HDL ratio	3.00 (2.63–3.76)	3.44 (2.78–4.03)	2.81 (2.56–3.29)	0.01
LDL-C:HDL-C ratio	1.76 (1.42–2.30)	2.08 (1.58–2.56)	1.60 (1.35–2.04)	0.01
Glucose, mmol/L	5.03 ± 0.48	5.13 ± 0.51	4.94 ± 0.44	0.01
Insulin, pmol/L	26.4 (17.3–39.9)	27.1 (16.9–42.5)	26.3 (18.2–37.7)	0.69
HOMA-IR	0.98 (0.07–5.30)	1.04 (0.63–1.63)	0.97 (0.62–1.41)	0.41
NEFA, μmol/L	416 (318–546)	388 (310–518)	427 (327–567)	0.01
CRP, mg/L	0.62 (0.29–1.46)	0.63 (0.31–1.43)	0.62 (0.28–1.52)	0.91
GGT, U/L	16.9 (14.0–22.7)	20.5 (16.2–27.5)	15.3 (13.2–19.0)	0.01
Uric acid, µmol/L	280 ± 68	323 ± 59	242 ± 51	0.01
Adiponectin, µg/mL	5.11 (2.48–9.07)	4.19 (2.22–6.02)	6.70 (2.93–11.38)	0.01
25-Hydroxyvitamin D, ng/mL	23.9 ± 11.3	23.4 ± 10.8	24.3 ± 11.7	0.50

*A/G fat ratio* android to gynoid fat ratio, *BMI* body mass index, *CRP* C-reactive protein, *F* female, *FFM* fat free mass, *FMI* fat mass index, *GGT* gamma-glutamyl transferase, *HC* hip circumference, *HDL-C* high density lipoprotein cholesterol, *LDL-C* low density lipoprotein cholesterol, *LMI* lean mass index, *M* male, *NEFA* non-esterified fatty acids, *TAG* triacylglycerol, *TC* total cholesterol, *VAT* visceral adipose tissue, *VAI* visceral adiposity index, *WC* waist circumference, *WHR* waist to hip ratio, *WHtR* waist to height ratio

^a^Data were analyzed by independent t tests and presented as mean ± SD or median (interquartile range); *p* ≤ 0.05 was considered significant

^b^Sample sizes differ as follows: Blood pressure n = 406 (M:187/F:219); body composition measures n = 370 (M:174/F:196); biochemistry n = 405 (M:188/F:217); insulin and HOMA-IR n = 272 (M:109/F:163); NEFA n = 362 (M:168/F:194); CRP n = 403 (M:188/F:215), GGT n = 330 (M:135/F:195); UA, adiponectin and 25-hydroxyvitamin D, n = 366 (M:172/F:194)

**Table 2:** Dietary intake and physical activity levels of the study participants

	All (n = 391)	Men (n = 179)	Women (n = 239)	*p* value^a^
*Dietary energy and macronutrient intake*				
Energy, MJ/day	8.50 ± 2.47	9.62 ± 2.51	7.56 ± 2.00	< 0.01
Total Fat, %TE	36.5 ± 8.6	34.6 ± 9.6	36.4 ± 7.8	0.82
SFA, %TE	13.0 ± 4.5	13.3 ± 5.2	12.8 ± 3.7	0.36
MUFA, %TE	13.7 ± 3.8	13.6 ± 4.1	13.8 ± 3.6	0.70
PUFA, %TE	6.17 ± 2.11	6.09 ± 2.33	6.24 ± 1.91	0.51
n-6 PUFA, %TE	5.66 ± 2.91	5.33 ± 3.03	5.93 ± 2.79	0.04
n-3 PUFA, %TE	0.86 ± 0.59	0.82 ± 0.49	0.90 ± 0.67	0.17
Trans fat, %TE	0.49 (0.34–0.68)	0.50 (0.35–0.72)	0.49 (0.33–0.63)	0.05
Protein, %TE	17.1 (14.8–20.2)	17.0 (14.5–20.4)	17.4 (15.0–19.7)	0.58
Carbohydrate, %TE	45.8 ± 10.9	45.4 ± 12.1	46.1 ± 9.8	0.52
Total Sugars, %TE	18.7 ± 6.6	17.7 ± 7.0	19.6 ± 6.0	0.01
Dietary Fibre (AOAC), g/day	24.3 ± 8.8	25.2 ± 8.9	23.5 ± 8.6	0.07
*Physical activity level*^b^				
Steps/day	8953 (6948–11,941)	8500 (6517–10,717)	9288 (7193–12,024)	0.02
Energy expended (kcal/day)	254 (157–431)	324 (195–524)	224 (141–349)	< 0.01
Percentage time per day spent				
Sedentary	69.8 ± 7.3	71.1 ± 7.4	68.9 ± 7.1	0.01
Performing light PA	25.5 ± 6.8	24.3 ± 6.6	26.3 ± 6.9	0.01
Performing moderate to vigorous PA	4.2 (2.7–6.2)	4.0 (2.6–6.1)	4.4 (2.7–6.3)	0.34

*AOAC* Association of Official Analytical Chemist, *MUFA* monounsaturated fatty acids, *PA* physical activity, *PUFA* polyunsaturated fatty acids, *SFA* saturated fatty acids, *%TE* % of total energy

^a^Differences between men and women were analyzed by independent t test and presented as mean ± SD or median (interquartile range); *p* ≤ 0.05 was considered significant

^b^Sample sizes differed as follows: Physical activity level n = 327 (M:126/F:201) and steps/day n = 309 (M:120/F:189)

For further analysis, the study cohort with dietary data was stratified according to dietary SFA intake expressed as %TE. Subjects in Q1 were selected to be within dietary recommendations for SFA (≤ 10%TE). General linear model (ANCOVA) was performed to investigate the impact of increasing intakes of dietary SFAs on subject characteristics, adjusting for age and sex. Post-hoc analyses with a Bonferroni correction were used to compare differences between the SFA%TE quartile groups. Results are presented as estimated marginal means ± SE for normally distributed and as median (interquartile range) for non-normally distributed variables in Table 5 and *p* ≤ 0.05 was considered significant.

## Results

### Study participants

**Fig. 1:**
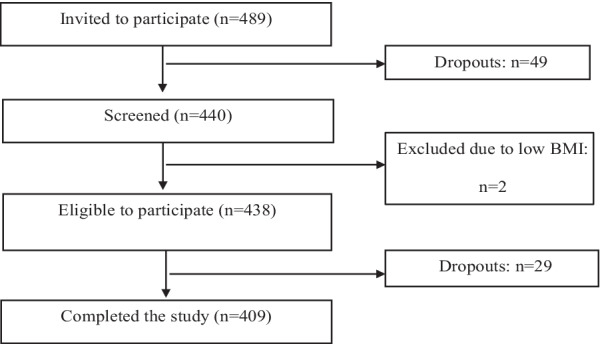
Flow chart of participants from the BODYCON study

A total of 438 healthy subjects were recruited, 29 of them dropped out between the screening and the main visit and 409 subjects (219 were women and 190 men) completed the study. The flow of participants in the study is shown in Fig. 1. The cohort had a mean age of 42 ± 16 years and median BMI of 23.5 (IQR 21.5–25.9) kg/m^2^. The main characteristics of the BODYCON study participants are shown in Table 1. Men (47%) and women (53%) were approximately equally distributed and matched for age in the study population. Compared with women, men had greater BMI, body weight, WC, WHR, WHtR, SBP, and DBP (*p* < 0.01 each). Women had significantly higher body fat, android fat percentage, gynoid fat percentage and fat mass (*p* ≤ 0.03), whilst men had a higher lean body mass, abdominal VAT mass, and android:gynoid (A/G) percentage fat ratio (*p* < 0.01 each). Moreover, men had higher fasting serum TAG, glucose, GGT and UA concentrations and TC: HDL-C ratio (*p* < 0.01 for all), while women had higher HDL-C, NEFA and adiponectin concentrations (*p* ≤ 0.01) (Table 1).

The dietary intakes (n = 391) and physical activity (n = 327) levels of the study participants are shown in Table 2. Within the cohort, 2 subjects were identified as under-reporters and 3 as over-reporters, with 13 further subjects excluded due to completion of < 3 days of dietary intake (n = 1) or did not provide a diet diary (n = 12). For the mean dietary intakes, men reported greater energy intakes (*p* < 0.01), but only trans-fat (%TE) intake was higher in men in terms of dietary macronutrients compared to women (*p* = 0.05), while women reported higher total sugar (%TE) and n-6 PUFA (%TE) intakes compared to men (*p* ≤ 0.04). Regarding physical activity levels, 82 subjects were excluded according to inclusion criteria for the physical activity analysis. Compared with men, women had higher daily step counts and spent a greater percentage of time during the day performing light physical activity (*p* ≤ 0.02). On average, men expended significantly more energy per day (approximately 100 kcal/day) performing physical activity compared with women (*p* < 0.01). The percentage of time spent performing moderate to vigorous physical activity daily was not different between the sexes (*p* = 0.34) (Table 2).

### Association between body composition, cardiometabolic disease risk markers and dietary macronutrients

Correlations between body composition measurements, CVD risk markers and dietary macronutrients in the whole group are shown in Table 3 and Additional file 1: Table 1 and according to sex in Additional file 1: Tables S2 and S3. In the whole group body fat mass was found to have weak positive correlations with SBP and DBP, while abdominal VAT mass and A/G fat ratio had moderate positive correlations with both SBP and DBP (*p* < 0.01). In addition, inverse moderate correlations were evident between HDL-C and several adiposity measurements, including abdominal VAT mass (*p* < 0.01). In contrast, moderate positive correlations were found between TAG, non-HDL-C, TC: HDL-C ratio and LDL-C: HDL-C ratio with abdominal VAT mass, android fat mass, android fat percentage and A/G fat ratio (*p* < 0.01 for each). There were also weak positive correlations between LDL-C with SFA (%TE) and trans-fat (%TE) (*p* < 0.01 for each). Weak correlations were found between dietary macronutrients and cardiometabolic disease risk markers, with SFA (%TE) intake positively associated with TC, LDL-C, non-HDL-C and NEFA (*p* ≤ 0.05), whereas carbohydrate (%TE) intake was negatively correlated with LDL-C (*p* < 0.01).

**Table 3:** Spearman’s correlation coefficients (r_s_) for the relationship between DXA body composition measurements, with CVD risk factors and dietary macronutrients

	Body fat, %	Fat mass, kg	Lean mass, kg	VAT, g	Android fat, kg	Android fat %	Gynoid fat %	A/G
*Blood pressure*, mmHg								
Systolic	−0.03	0.15**	0.33**	0.40**	0.25**	0.14**	−0.15**	0.33**
Diastolic	0.13*	0.29**	0.21**	0.44**	0.37**	0.31**	−0.003	0.39**
Pulse pressure	−0.14**	−0.02	0.25**	0.16**	0.03	−0.05	−0.19**	0.11*
*Biochemistry*								
TC, mmol/L	0.19**	0.17**	−0.11*	0.25**	0.23**	0.23**	0.13*	0.20**
TAG, mmol/L	0.21**	0.35**	0.18**	0.46**	0.42**	0.38**	0.06	0.43**
HDL-C, mmol/L	−0.02	−0.23**	−0.35**	−0.35**	−0.29**	−0.20**	0.11*	−0.34**
LDL-C, mmol/L	0.17**	0.20**	−0.02	0.32**	0.27**	0.25**	0.09	0.26**
Non-HDL-C, mmol/L	0.22**	0.27**	0.02	0.41**	0.36**	0.33**	0.10	0.35**
TC: HDL ratio	0.18**	0.37**	0.24**	0.55**	0.47**	0.39**	−0.001	0.50**
LDL-C: HDL-C ratio	0.16**	0.33**	0.23**	0.51**	0.43**	0.35**	−0.01	0.46**
NEFA, μmol/L	0.20**	0.12*	−0.17**	0.04	0.10*	0.15**	0.20**	−0.02
Glucose, mmol/L	0.07	0.23**	0.22**	0.41**	0.31**	0.23**	−0.05	0.35**
Insulin, pmol/L	0.35**	0.41**	0.02	0.34**	0.41**	0.42**	0.24**	0.32**
HOMA-IR	0.34**	0.42**	0.06	0.38**	0.43**	0.42**	0.21**	0.34**
CRP, mg/L	0.36**	0.41**	0.001	0.29**	0.39**	0.41**	0.27**	0.27**
GGT, U/L	−0.09	0.11*	0.35**	0.37**	0.22**	0.11	−0.22**	0.36**
Uric acid, µmol/L	−0.16**	0.13*	0.53**	0.43**	0.29**	0.13*	−0.35**	0.51**
Adiponectin, µg/mL	0.18**	0.02	−0.25**	−0.14**	−0.05	0.02	0.25**	−0.21
Total 25(OH)D, ng/mL	−0.16**	−0.14**	0.05	−0.12*	−0.08	−0.11*	−0.15**	−0.14**
*Dietary intake*								
Total fat, %TE	0.02	0.01	−0.03	0.01	0.01	0.01	0.02	0.01
SFA %TE	0.04	0.06	0.01	0.08	0.06	0.04	0.03	0.04
MUFA, %TE	0.01	−0.01	−0.05	−0.02	−0.01	−0.004	0.02	−0.01
PUFA, %TE	−0.07	−0.15**	−0.09	−0.13*	−0.16**	−0.13*	−0.04	−0.14**
n-6 PUFA, %TE	−0.08	−0.16**	−0.11*	−0.17**	−0.19**	−0.15**	−0.02	−0.18**
n-3 PUFA, %TE	−0.004	−0.05	−0.08	0.01	−0.04	−0.01	0.02	−0.03
Trans fat, %TE	0.07	0.11*	0.07	0.16**	0.13**	0.11*	0.04	0.12*
Protein, %TE	−0.03	−0.003	0.07	−0.04	−0.02	−0.05	−0.03	−0.07
Carbohydrate, %TE	0.02	0.003	−0.05	−0.03	0.001	0.02	0.03	0.01
Fibre (AOAC), g/day	−0.22**	−0.14**	0.21**	−0.06	−0.13*	−0.21**	−0.21**	−0.07
Total Sugars, %TE	0.03	−0.01	−0.11*	−0.14**	−0.04	−0.04	0.07	−0.11*

Data analysed by Spearman’s correlations

*AOAC* association of analytical chemists, *A/G* android to gynoid ratio, *CRP* C-reactive protein, *GGT* gamma-glutamyl transferase, *HDL-C* high density lipoprotein cholesterol, *HOMA-IR* homeostatic model assessment for insulin resistance, *LDL-C* low density lipoprotein cholesterol, *MUFA* monounsaturated fatty acids, *NEFA* non-esterified fatty acids, *PUFA* polyunsaturated fatty acids, *SFA* saturated fatty acids, *TC* total cholesterol, *TAG* triacylglycerol, *%TE* % of total energy, *VAT* abdominal visceral adipose tissue, *total 25(OH)D*: 25-hydroxyvitamin D

*Significant differences at the 0.05 level

**Significant differences at the 0.01 level

For abdominal VAT mass, moderate positive correlations were found with insulin, HOMA-IR, glucose, CRP and uric acid, while there were weak, negative correlations with adiponectin and 25-hydroxyvitamin D levels (*p* ≤ 0.05). Regarding the association between diet and body composition, we observed weak correlations. n-6 PUFA (%TE) intake was negatively correlated with abdominal VAT mass, while trans-fat (%TE) intake was positively correlated (*p* < 0.01) (Table 3).

After stratifying the group according to sex, a few sex-specific associations were observed. Body fat mass and abdominal VAT mass were found to have weak to moderate positive correlations with both SBP and DBP in women, while only with DBP in men (*p* < 0.01). In addition, inverse moderate correlations were evident between HDL-C and several adiposity measurements, including abdominal VAT mass in men (*p* < 0.01), while there were weak inverse correlations between HDL-C and percentage body fat, fat mass and android fat percentage in women (*p* < 0.05). Abdominal VAT mass was negatively correlated with n-6 PUFA (%TE) in men (*p* < 0.01), whilst in women there was a weak inverse correlation with carbohydrate (%TE) intake (*p* < 0.05) (Additional file 1: S3).

### Stepwise multivariate regression analysis

The standardized regression coefficients, adjusted r^2^ and *p* values for the stepwise multivariate regression analysis are shown in Table 4. Only SFA (%TE) intake, abdominal VAT mass, total fat (%TE) and carbohydrate (%TE) intakes were found to be independently associated with fasting LDL-C, explaining 18.3% of the variability in this established CVD risk marker. Of these variables, 9% of this variability was explained by SFA (%TE) intake and 7% by abdominal VAT mass (Table 4).

**Table 4:** Stepwise multivariate linear regression analysis exploring the relation between dietary macronutrients, body composition and biochemical variables with LDL-C and abdominal VAT

Dependent variable	Independent variable	Standardized coefficient	Adjusted r^b^	*p* value
LDL-C^a^	SFA %TE	0.297	0.085	< 0.01
	and Abdominal VAT	0.277	0.160	< 0.01
	and Carbohydrate %TE	−0.157	0.172	0.013
	and Fat %TE	−0.261	0.183	0.017
Abdominal VAT^b^	TC: HDL-C	0.572	0.325	< 0.01
	and DBP	0.314	0.415	< 0.01
	and GGT	0.253	0.475	< 0.01
	and HOMA-IR	0.240	0.518	< 0.01
	and Sex (female)	−0.237	0.565	< 0.01
	and Age	0.222	0.606	< 0.01
	and HDL-C	−0.202	0.625	0.001
	and Uric acid	0.157	0.636	0.005

*GGT* gamma-glutamyl transferase, *HDL-C* high density lipoprotein cholesterol, *HOMA-IR* homeostatic model assessment for insulin resistance, *LDL-C* low-density lipoprotein cholesterol, *SFA* saturated fatty acid, *TC* total cholesterol, *%TE* % of total energy, *VAT* visceral adipose tissue

^a^Variables included in the analysis: BMI, body fat %, fat mass, abdominal VAT, android fat mass, android fat %, A/G fat ratio, WC, HC, WHR, WHtR, fat %TE, SFA %TE, trans-fat %TE, CHO %TE

^b^Variables included in the analysis: age, sex, TC, TAG, HDL-C, LDL-C, non-HDL-C, TC: HDL-C, LDL-C: HDL-C, glucose, 25(OH)D, CRP, GGT, UA, adiponectin, insulin, HOMA-IR, SBP, DBP, PP, PUFA %TE, n-6 PUFA %TE, trans-fat %TE, total sugars %TE

The TC: HDL-C ratio, DBP, GGT, HOMA-IR, sex, age, HDL-C and uric acid were independently associated with abdominal VAT mass and, together, these variables explained 64% of the variability in abdominal VAT mass. This analysis showed that TC: HDL-C ratio alone explained 33% of the variability in the mass of this fat depot.

### Subject characteristics according to quartiles of dietary SFA (%TE) intake

There were no significant differences in mean body weight (*p* = 0.10) or BMI (*p* = 0.20) across quartiles (Q) of increasing %TE from SFA (Table 5). However, android lean mass was found to be 7% higher in Q3 compared with Q1 (*p* = 0.02). Other anthropometric measures were not different across the quartiles of SFA%TE intake.

**Table 5:** Participant’s characteristics according to quartiles of dietary saturated fatty acid (%TE) intake

Characteristics^2^	Q1 (n = 78)	Q2 (n = 101)	Q3 (n = 109)	Q4 (n = 103)	*p* value^1^
	(1.9–10.0%TE)	(10.1–11.9%TE)	(12.0–14.8%TE)	(14.9–38.7%TE)	
Weight, kg	68.4 ± 1.3	69.5 ± 1.2	72.5 ± 1.1	69.9 ± 1.2	0.10
BMI, kg/m^2^	23.0 (20.8–25.4)	23.2 (21.4–25.5)	24.1 (22.0–27.0)	23.6 (21.5–25.7)	0.20
WC, cm	82.5 ± 1.1	83.5 ± 1.0	85.4 ± 1.0	83.3 ± 1.0	0.21
HC, cm	100 ± 1	100 ± 1	103 ± 1	101 ± 1	0.06
WHR	0.83 ± 0.01	0.83 ± 0.01	0.83 ± 0.01	0.83 ± 0.01	0.99
WHtR	0.49 ± 0.01	0.49 ± 0.01	0.50 ± 0.01	0.49 ± 0.01	0.42
*Blood pressure*, mmHg					
SBP	120 ± 2^ab^	121 ± 1^ab^	117 ± 1^b^	123 ± 2^a^	0.01
DBP	71 ± 1	72 ± 1	71 ± 1	72 ± 1	0.83
Pulse pressure	48 ± 1^ab^	49 ± 1^a^	45 ± 1^b^	50 ± 1^a^	< 0.01
*Body composition measures*					
Body fat, %	28.4 ± 0.8	28.3 ± 0.7	29.1 ± 0.7	27.6 ± 0.7	0.53
Android fat, %	30.5 ± 1.4	30.8 ± 1.2	31.6 ± 1.2	29.6 ± 1.2	0.68
Gynoid fat, %	32.5 ± 0.8	32.2 ± 0.7	33.2 ± 0.7	31.6 ± 0.7	0.47
A/G fat ratio	0.94 ± 0.03	0.96 ± 0.02	0.97 ± 0.02	0.94 ± 0.02	0.79
Fat mass, kg	17.7 (12.3–25.3)	17.9 (14.5–25.0)	20.2 (16.1–25.4)	19.0 (14.3–24.0)	0.21
Lean mass, kg	46.8 ± 0.8	48.0 ± 0.7	49.2 ± 0.7	48.3 ± 0.7	0.12
Android fat mass, kg	1.63 ± 0.12	1.57 ± 0.10	1.72 ± 0.10	1.54 ± 0.10	0.58
Android lean mass, kg	3.19 ± 0.06^a^	3.30 ± 0.05^ab^	3.42 ± 0.05^b^	3.28 ± 0.05^ab^	0.02
Abdominal VAT, g	562 ± 56	582 ± 50	651 ± 48	562 ± 49	0.53
*Indexes*					
VAI	1.05 ± 0.08	1.02 ± 0.07	1.06 ± 0.07	0.94 ± 0.07	0.61
*Biochemistry*					
TC, mmol/L	5.17 ± 0.11^ab^	4.91 ± 0.10^a^	5.10 ± 0.09^ab^	5.39 ± 0.09^b^	0.01
TAG, mmol/L	0.84 (0.67–1.18)	0.82 (0.65–1.07)	0.84 (0.66–1.28)	0.84 (0.67–1.11)	0.59
HDL-C, mmol/L	1.67 ± 0.04	1.61 ± 0.04	1.61 ± 0.04	1.73 ± 0.04	0.05
LDL-C, mmol/L	3.00 ± 1.00^ab^	2.87 ± 0.08^a^	3.04 ± 0.08^ab^	3.23 ± 0.08^b^	0.03
Non-HDL, mmol/L	3.50 ± 0.10^ab^	3.31 ± 0.09^a^	3.49 ± 0.09^ab^	3.66 ± 0.09^b^	0.05
TC: HDL-C	2.95 (2.65–3.71)	2.94 (2.61–3.44)	3.03 (2.61–3.81)	3.15 (2.61–3.83)	0.61
LDL-C: HDL-C	1.69 (1.43–2.23)	1.71 (1.42–2.13)	1.82 (1.41–2.42)	1.90 (1.41–2.40)	0.50
NEFA, μmol/L	390 (327–534)	403 (294–500)	441 (310–560)	456 (340–598)	0.36
Glucose, mmol/L	4.98 ± 0.05	5.08 ± 0.04	5.01 ± 0.04	5.02 ± 0.04	0.49
CRP, mg/L	0.49 (0.25–1.29)	0.59 (0.27–1.37)	0.66 (0.38–1.54)	0.66 (0.29–1.69)	0.79
Uric acid, µmol/L	270 ± 7	275 ± 6	289 ± 6	282 ± 6	0.14
Adiponectin, µg/mL	6.03 (2.50–10.62)^a^	4.93 (2.39–7.99)^ab^	4.09 (1.99–8.12)^b^	5.45 (3.11–9.87)^ab^	0.03
Total 25-Hydroxyvitamin D, ng/mL	22.3 ± 1.3	24.3 ± 1.2	24.3 ± 1.1	24.2 ± 1.2	0.63
Insulin, pmol/L	26.0 (20.3–37.5)	26.5 (17.3–38.6)	22.4 (15.9–40.1)	28.7 (18.3–42.7)	0.61
HOMA-IR	1.00 (0.68–1.44)	1.00 (0.65–1.53)	0.85 (0.58–1.44)	1.15 (0.66–1.63)	0.60
*Dietary intake*					
Energy, kcal/day	1906 ± 61	2045 ± 54	2055 ± 51	2092 ± 53	0.13
Energy, MJ/day	7.98 ± 0.26	8.56 ± 0.22	8.60 ± 0.22	8.75 ± 0.22	0.13
Total fat, %TE	28.2 ± 0.7^a^	34.3 ± 0.6^b^	36.5 ± 0.6^b^	44.9 ± 0.6^c^	< 0.01
MUFA, %TE	11.0 ± 0.4^b^	13.3 ± 0.3^a^	13.8 ± 0.3^a^	16.1 ± 0.3^c^	< 0.01
PUFA, %TE	5.97 ± 0.24	6.39 ± 0.21	6.04 ± 0.20	6.24 ± 0.21	0.52
n-6 PUFA, %TE	5.64 ± 0.33^ab^	6.48 ± 0.29^a^	5.53 ± 0.28^ab^	5.00 ± 0.28^b^	< 0.01
n-3 PUFA, %TE	0.84 ± 0.07^ab^	0.87 ± 0.06^ab^	0.76 ± 0.06^a^	0.98 ± 0.06^b^	0.06
PUFA/SFA	0.81 ± 0.03^a^	0.58 ± 0.03^b^	0.46 ± 0.03^c^	0.34 ± 0.03^d^	< 0.01
MUFA/SFA	1.44 ± 0.04^a^	1.21 ± 0.03^b^	1.04 ± 0.03^c^	0.89 ± 0.03^d^	< 0.01
Trans fat, %TE	0.30 (0.23–0.40)^a^	0.41 (0.31–0.54)^b^	0.53 (0.43–0.68)^c^	0.76 (0.56–0.96)^d^	< 0.01
Protein, %TE	17.6 (14.7–20.5)	18.1 (15.7–20.9)	16.5 (14.5–19.2)	16.5 (14.5–19.8)	0.44
Carbohydrate, %TE	54.1 ± 1.1^b^	47.3 ± 0.9^a^	46.1 ± 0.9^a^	37.7 ± 0.9^c^	< 0.01
Fiber (AOAC), g/day	27.3 ± 1.0^a^	26.3 ± 0.9^ab^	23.8 ± 0.8^b^	20.5 ± 0.8^c^	< 0.01
Total Sugars, %TE	19.6 ± 0.7	18.9 ± 0.6	19.4 ± 0.6	17.1 ± 0.6	0.03
*Physical activity level*					
Steps/day	9786 (7583–12,573)	9153 (6883–11,523)	8937 (6876–11,973)	8177 (6715–11,206)	0.25
Energy expended (kcal/day)	296 (164–525)	265 (162–450)	249 (146–385)	231 (144–330)	0.16
Percentage time spent per day					
Sedentary	70.0 ± 0.9	69.7 ± 0.8	69.8 ± 0.8	70.1 ± 0.8	0.98
Performing light PA	25.0 ± 0.8	25.7 ± 0.7	25.6 ± 0.7	25.4 ± 0.7	0.89
Performing moderate to vigorous PA	4.6 (2.9–6.8)	4.2 (3.1–6.4)	4.0 (2.5–6.6)	4.0 (2.5–5.6)	0.64

*AOAC* Association of Official Analytical Chemist, *CRP* C-reactive protein, *DBP* diastolic blood pressure, *HC* hip circumference, *HDL-C* high density lipoprotein cholesterol, *LDL-C* low density lipoprotein cholesterol, *MUFA* monounsaturated fatty acids, *NEFA* non-esterified fatty acids, *PA* physical activity, *PUFA* polyunsaturated fatty acids, *SFA* saturated fatty acids, *SBP* systolic blood pressure, *TC* total cholesterol, *TAG* triacylglycerol, *UA* uric acid, *VAI* visceral adiposity index, *WC* waist circumference, *WHR* waist to hip ratio, *WHtR* waist to height ratio

^1^Data were analysed by ANCOVA with age and sex as covariates and presented as estimated mariginal means ± SE or median (interquartile range); *p* ≤ 0.05 considered significant

^2^Sample sizes differ as follows: Blood pressure, Q1 n = 77, Q2 n = 101, Q3 n = 107, Q4 n = 103; body composition measures, Q1 n = 72, Q2 n = 91, Q3 n = 98, Q4 n = 94; VAI, Q1 n = 78, Q2 n = 99, Q3 n = 103, Q4 n = 103; biochemistry, Q1 n = 78, Q2 n = 100, Q3 n = 106, Q4 n = 103; NEFA, Q1 n = 72, Q2 n = 89, Q3 n = 92, Q4 n = 94; CRP, Q1 n = 77, Q2 n = 99, Q3 n = 106, Q4 n = 103; UA, adiponectin and 25-hydroxyvitamin D, Q1 n = 72, Q2 n = 90, Q3 n = 95, Q4 n = 94; insulin and HOMA-IR, Q1 n = 52, Q2 n = 71, Q3 n = 67, Q4 n = 69; dietary intake, Q1 n = 78, Q2 n = 101, Q3 n = 109, Q4 n = 103; physical activity, Q1 n = 69, Q2 n = 80, Q3 n = 84, Q4 n = 85; steps/day, Q1 n = 66, Q2 n = 77, Q3 n = 77, Q4 n = 81

Significant differences in several cardiometabolic disease risk markers were also evident across increasing quartiles of SFA (%TE) intake. SBP and pulse pressure were higher in Q4 compared to Q3 (*p* ≤ 0.01). TC, LDL-C and non-HDL-C levels were 9%, 12% and 10% higher in Q4 than Q2, respectively (*p* ≤ 0.05). Regarding dietary intakes, subjects in Q4 reported higher total fat, MUFA and trans-fat (%TE) than other quartiles (*p* < 0.01 each) and lower n-6 PUFA (%TE) intake than Q2 (*p* < 0.01). Carbohydrate (%TE) and fiber (g/day) intakes were lowest in Q4 compared to other quartiles (*p* < 0.01 each) (Table 5).

## Discussion

The present study investigated the associations between dietary SFA intake, cardiometabolic disease risk markers and body composition to determine whether body fat distribution contributed to the relationship between SFA and LDL-C in a group of healthy adults. Although our study does not establish cause and effect relationships due to its cross-sectional nature, we observed interesting and novel associations. In particular, dietary SFA, total fat and carbohydrate intakes and abdominal VAT mass were independently associated with LDL-C and found to explain 18.3% of the variability. However, SFA intake was not related to abdominal VAT mass. Furthermore, stratification according to quartiles of dietary SFA intake did not reveal dose-dependent relationships with LDL-C, TC, non-HDL-C, blood pressure or android lean mass.

The replacement of dietary SFA with unsaturated fatty acids (n-6 PUFA and MUFA) is associated with beneficial effects on the fasting blood lipid profiles [37]. In the PURE cross-sectional study, which included 104 486 men and women aged 30–70 years from 18 countries, dietary SFA intake was positively related with LDL-C and replacing 5%TE of dietary SFA with PUFA and MUFA was associated with lower LDL-C concentrations (between 0.02 and 0.18 mmol/L) using a multivariable nutrient density model [38]. In agreement with previous studies, we also observed an independent positive association between LDL-C and dietary SFA, with dietary SFAs explaining 9% of the variability in LDL-C response between individuals. However, after stratifying data by SFA intake, we did not observe a linear relationship between increasing SFA intakes and LDL-C, with differences only evident in TC, LDL-C and non-HDL-C concentrations between Q2 and Q4. The lack of a dose-dependent relationship between SFA intake and LDL-C may reflect the use of age and sex as co-variates in the ANCOVA analysis, which are both important non-modifiable determinants of LDL-C concentrations [39, 40]. Furthermore, the association of dietary SFA with CVD risk has been proposed to be dependent on the food source and the type of individual SFA rather than the amount of the SFA. For example, although high in SFA, dairy have been reported to have neutral or positive effects on CVD risk markers [41], whereas palmitic acid has been reported to be more atherogenic than stearic acid [42]. Therefore, determining total dietary SFA intake in the current study may have influenced the strength of the relationship with fasting LDL-C due to the differences in frequency of dairy product and/or individual SFA consumption within the quartile groups [41]. Interestingly, n-6 PUFA intake was considerably higher in Q2 compared to Q4, which may have also influenced blood cholesterol levels. Furthermore, high intakes of plant-based MUFA are associated with lower LDL-C concentrations [43] and in this cohort those with the highest SFA intakes also had higher total fat and MUFA intakes. However, this would not necessarily represent a higher intake of plant-based MUFA-rich foods and oils, as animal products are also a rich source of both SFAs and MUFAs. Similarly, increasing trans-fat intake across quartiles of dietary SFA might be due to the major dietary sources of trans-fats being high in dietary SFA [44]. Participants consuming on average 8%TE SFA (Q1) also had the highest carbohydrate intake (54.1%TE), which exceeded the recommended intake of 45–50%TE. It is clear from literature that replacing SFA with carbohydrate can increase fasting TAG and lower HDL-C concentrations in some population sub-groups [45, 46]. Moreover, Hooper et al. [4] reported no effect of replacing SFA with carbohydrate on CVD events and mortality in a systematic review and meta-analysis of 6 randomized controlled trials, while Schwab et al. [2] reported an increased risk of CVD outcomes in a systematic review of prospective cohort studies. Therefore, our contradictory results may be due to the higher carbohydrate intakes in the quartile which met the SFA dietary recommendation for CVD risk reduction (Q1). Interestingly, although Q2 consumed more carbohydrate than Q4, their fiber consumption was higher which might have positively influenced blood cholesterol concentrations [47]. This could suggest that the positive association of high-fat, low SFA diets on lipid risk markers might also be dependent on other dietary macronutrients and overall dietary pattern [48].

The observation that dietary SFA intake was independently associated with the fasting LDL-C concentration may be related to the impact of dietary fatty acids on LDL particle clearance. Animal and in vitro studies have suggested that dietary SFAs increase LDL-C via a downregulation in the number and expression of the hepatic LDL receptor (LDL-R) [49–51]. Although the mechanisms are still not totally understood, animal studies have provided evidence that dietary fat quality affects the LDL-R at the molecular level potentially through its effect on mRNA expression [52]. A possible explanation is that dietary SFAs lower the esterification of cholesterol in the liver by inhibiting the cholesterol esterifying enzyme acyl-CoA: cholesterol acyltransferase (ACAT), leading to increased free cholesterol accumulation which then suppress the activity of transcription factors such as sterol regulatory element-binding proteins and liver X receptor, downregulating LDL-R gene expression [52–54]. In contrast, a recent study showed an increase in hepatic expression of ACAT-2 in mice fed short-term with a high SFA diet and in HepG2 cells treated with 0.5 mmol/l and 1 mmol/l palmitic acid for 14 h [55]. Furthermore, it has been argued in another study in hamsters that increased ACAT activity may result in the formation of larger cholesterol ester enriched very low-density lipoprotein (VLDL) particles which may be the reason for increased LDL-C concentrations. The authors discussed that the effect of dietary fat composition on circulating cholesterol concentration might be via increased hepatic lipoprotein secretion rather than clearance [56]. Findings from an in vitro study conducted in HepG2 cells suggested that enrichment of VLDL particles with apoE following a meal rich in dietary SFA could lead to greater competition with LDL for hepatic LDL-R uptake [57]. However, these findings are from cell or animal studies, so there is a need for further studies in humans to understand the mechanisms behind the association between dietary SFAs and LDL-C concentrations.

Higher intakes of dietary SFAs have been suggested to be associated with abdominal fat accumulation, increasing CVD risk [23]. In contrast to some studies [23, 25, 58–60], we found no relationship between body fat distribution including abdominal VAT mass and SFA intake in this study. However, our findings are consistent with Greenfield et al. [61] who reported a lack of association between adiposity and dietary fat composition in their cross-sectional study in 334 female twins. This discrepancy between studies might be due to the difference in participant characteristics, study design or methods used for dietary and body composition assessments. Surprisingly, android lean mass was highest in Q3. This finding might be associated with their low carbohydrate, high SFA diet, which has previously been reported to increase lean mass, but this has only been observed in diets with high protein intakes (20–30%TE) [62–64]. Therefore, although abdominal VAT mass explained 7% of variability in LDL-C, it was not found to be different across dietary SFA quartiles. These findings suggest that dietary SFAs and abdominal VAT may impact on LDL-C via different mechanisms.

Body fat distribution, especially abdominal VAT accumulation, has been associated with CVD risk independent of BMI, while gynoid fat is thought to be protective against metabolic diseases [65, 66]. In the current study, fasting blood lipids (TC, TAG and LDL-C) which are established CVD risk markers, were positively associated with body fat distribution measures, including abdominal VAT mass, which confirms previous studies [67]. Furthermore, we found the TC: HDL-C ratio to explain the largest proportion of variability in abdominal VAT mass between individuals, which highlights the importance of body fat distribution in relation to CVD risk.

One proposed link between abdominal VAT and CVD is chronic and systemic inflammation, which may occur due to impaired adipocyte differentiation [68]. People with VAT accumulation have been shown to have hypertrophic dysfunctional adipocytes which release pro-inflammatory factors. Due to the location of abdominal VAT, these pro-inflammatory factors can enter the liver via the portal vein and increase glucose production leading to insulin resistance, which plays a role in the development of CVD [69]. In agreement, our study showed independent associations between CRP and HOMA-IR with abdominal VAT mass, supporting previous studies showing that increased abdominal VAT leads to development of pro-inflammatory state and insulin resistance [70]. Moreover, adipocyte hypertrophy is related to decreased adiponectin levels which has been associated with increased CVD incidence [71, 72]. In line with this, in our study, adiponectin levels were negatively correlated with abdominal VAT mass and were higher in women, who were shown to have lower abdominal VAT mass compared to men. Therefore, our findings lend support to the previously reported potential mechanisms for abdominal VAT mass and CVD risk.

Strengths of this study include the large sample size, the use of DXA scans to accurately measure body fat distribution and the use of detailed dietary and physical activity assessment. Moreover, compared to the results from the current National Diet and Nutrition Survey, the dietary intake of our cohort compared closely with this representative UK population [73]. Several limitations need consideration. First, our study cannot investigate cause and effect relationship due to its observational, cross-sectional design. Furthermore, dietary intake was self-reported using a 4-day weighed food diary, therefore measurement errors are inevitable. A further limitation is that participants can under and overestimate their food intake. We have tried to address this limitation by removing the under (n = 2) and over (n = 3) reporters from the dataset for dietary analysis. In addition, as it is not always possible to exactly match the food from volunteer’s diet diary with the food composition databases available, this may have influenced the dietary analysis data. Moreover, as limited data are available for n-3 and n-6 PUFA on the current UK food composition databases, these dietary data should be interpreted with caution. Furthermore, dietary SFA was assessed as a single nutrient instead of the food matrix (e.g., dairy and red meat), type (e.g., palmitic and stearic acid) or source (e.g., animal or plant sources) of SFA, which may modify its effect on disease risk markers. Lastly, our study attracted individuals with a predominately normal BMI and higher physical activity level than the average UK population, therefore, it may be difficult to translate our results to the general population.

In conclusion, the findings from this cross-sectional study indicate that both dietary SFA (%TE) and abdominal VAT mass were important determinants of the fasting LDL-C concentration. However, the lack of dose dependent relationships between quartiles of dietary SFA intake with abdominal VAT mass and LDL-C suggests that different mechanisms of action may exist for their impact on LDL. Therefore, further studies are needed to determine the impact of the types and sources of dietary SFA, and their relationship to abdominal obesity and CVD risk.

## Supplementary Information

**Additional file 1: Table S1.** Spearman’s correlation coefficients (r_s_) for the relationship between circulating cardiovascular disease risk markers and dietary macronutrient intakes in the whole group. **Table S2.** Spearman’s correlation coefficients (r_s_) for the relationship between circulating cardiovascular disease risk markers and dietary macronutrient intakes in men and women. **Table S3.** Spearman’s correlation coefficients (r_s_) for the relationship between DXA body composition measurements with CVD risk factors and dietary macronutrients in men and women.

## Data Availability

The datasets used and/or analysed during the current study are available from the corresponding author on reasonable request.

## Abbreviations

ACAT: Acyl-CoA: cholesterol acyltransferase
A/G fat ratio: Android to gynoid fat ratio
BMI: Body mass index
CRP: C-reactive protein
CVD: Cardiovascular disease
DBP: Diastolic blood pressure
DXA: Dual x-ray absorptiometry
EE_PA_: Mean energy expenditure from physical activity
GGT: Gamma-glutamyl transferase
HDL-C: High-density lipoprotein cholesterol
HOMA-IR: Homeostatic model assessment for insulin resistance
LDL-C: Low density lipoprotein cholesterol
MUFA: Monounsaturated fatty acids
NEFA: Non-esterified fatty acids
PUFA: Polyunsaturated fatty acids
SFA: Saturated fatty acid
SBP: Systolic blood pressure
TAG: Triacylglycerol
TC: Total cholesterol
VAT: Visceral adipose tissue
VAI: Visceral adiposity index
VLDL: Very low density lipoprotein
WC: Waist circumference
WHR: Waist to hip ratio
WHtR: Waist to height ratio
25(OH)D: 25 Hydroxy vitamin D
