# Supplementary material for: Association between dietary saturated fat with cardiovascular disease risk markers and body composition in healthy adults: findings from the cross-sectional BODYCON study
Source: Nutr Metab (Lond). 2022 Mar 3;19:15. doi: 10.1186/s12986-022-00650-y (PMC8896371; doi:10.1186/s12986-022-00650-y)
Supplement: Supplementary file 1 — Additional file 1: Table S1. Spearman’s correlation coefficients (rs) for the relationship between circulating cardiovascular disease risk markers and dietary macronutrient intakes in the whole group. Table S2. Spearman’s correlation coefficients (rs) for the relationship between circulating cardiovascular disease risk markers and dietary macronutrient intakes in men and women. Table S3. Spearman’s correlation coefficients (rs) for the relationship between DXA body composition measurements with CVD risk factors and dietary macronutrients in men and women. [file 12986_2022_650_MOESM1_ESM.docx]

**Association between dietary saturated fat with cardiovascular disease risk markers and body composition in healthy adults- Findings from the cross-sectional BODYCON study, Ozen et al.**

**SUPPLEMENTAL TABLES**

**Supplemental Table 1** Spearman’s correlation coefficients (r_s_) for the relationship between circulating cardiovascular disease risk markers and dietary macronutrient intakes in the whole group^1,2^

|  | TC | LDL-C | HDL-C | TAG | Non-HDL-C | TC:HDL-C ratio | LDL-C: HDL-C ratio | NEFA | Glucose | Insulin | CRP |
| --- | --- | --- | --- | --- | --- | --- | --- | --- | --- | --- | --- |
| Fat, %TE | 0.13* | 0.11* | 0.13* | -0.07 | 0.09 | -0.02 | -0.004 | 0.09 | 0.04 | -0.04 | 0.03 |
| SFA, %TE | 0.20** | 0.19** | 0.09 | -0.01 | 0.18** | 0.06 | 0.08 | 0.13* | 0.08 | 0.001 | 0.07 |
| MUFA, %TE | 0.09 | 0.08 | 0.11* | -0.07 | 0.06 | -0.03 | -0.01 | 0.04 | -0.004 | -0.03 | 0.01 |
| PUFA, %TE | 0.02 | -0.01 | 0.14** | -0.08 | -0.03 | -0.11* | -0.10 | -0.01 | -0.04 | -0.12* | -0.06 |
| n-3 PUFA, %TE | 0.10* | 0.08 | 0.09 | -0.02 | 0.06 | -0.02 | -0.01 | 0.07 | 0.04 | 0.02 | -0.02 |
| n-6 PUFA, %TE | 0.01 | -0.02 | 0.13* | -0.06 | -0.04 | -0.12* | -0.10* | -0.07 | -0.01 | -0.23** | 0.004 |
| Trans-fat, %TE | 0.19** | 0.19** | 0.03 | 0.04 | 0.20** | 0.13* | 0.13* | 0.11* | 0.10 | -0.01 | 0.08 |
| Protein, %TE | 0.08 | 0.07 | 0.09 | -0.02 | 0.05 | -0.003 | 0.01 | 0.03 | 0.03 | 0.05 | 0.08 |
| Carbohydrate, %TE | -0.23** | -0.19** | -0.18** | 0.02 | -0.18** | -0.01 | -0.03 | -0.06 | -0.12* | -0.01 | -0.07 |

^1^Data analysed by Spearman’s correlations

*Significant differences at the 0.05 level

**Significant differences at the 0.01 level

^2^Abbreviations: CRP: C-reactive protein; HDL-C: high density lipoprotein cholesterol; LDL-C: low density lipoprotein cholesterol; MUFA: monounsaturated fatty acids; NEFA: non-esterified fatty acids; PUFA: polyunsaturated fatty acids; SFA: saturated fatty acids; TC: total cholesterol; TAG: triacylglycerol; %TE: % of total energy.

**Supplemental Table 2** Spearman’s correlation coefficients (r_s_) for the relationship between circulating cardiovascular disease risk markers and dietary macronutrient intakes in men and women ^1,2^

|  | TC | LDL-C | HDL-C | TAG | Non-HDL-C | TC:HDL-C ratio | LDL-C: HDL-C ratio | NEFA | Glucose | Insulin | CRP |
| --- | --- | --- | --- | --- | --- | --- | --- | --- | --- | --- | --- |
| Fat, %TE |  |  |  |  |  |  |  |  |  |  |  |
| Women | 0.14* | 0.12 | 0.11 | 0.01 | 0.12 | 0.002 | 0.01 | 0.08 | 0.12 | -0.04 | 0.08 |
| Men | 0.10 | 0.10 | 0.13 | -0.14 | 0.08 | -0.01 | 0.01 | 0.09 | -0.03 | -0.06 | -0.03 |
| SFA, %TE |  |  |  |  |  |  |  |  |  |  |  |
| Women | 0.14* | 0.14* | 0.07 | 0.09 | 0.14* | 0.05 | 0.06 | 0.12 | 0.16* | 0.04 | 0.09 |
| Men | 0.24** | 0.25** | 0.14 | -0.11 | 0.22** | 0.10 | 0.13 | 0.13 | 0.01 | -0.08 | 0.05 |
| MUFA, %TE |  |  |  |  |  |  |  |  |  |  |  |
| Women | 0.07 | 0.07 | 0.02 | -0.01 | 0.06 | 0.03 | 0.03 | -0.02 | 0.04 | -0.05 | 0.02 |
| Men | 0.09 | 0.09 | 0.16* | -0.13 | 0.05 | -0.05 | -0.02 | 0.08 | -0.03 | -0.02 | -0.01 |
| PUFA, %TE |  |  |  |  |  |  |  |  |  |  |  |
| Women | 0.04 | 0.03 | 0.09 | -0.05 | 0.01 | -0.08 | -0.06 | -0.06 | -0.03 | -0.13 | -0.01 |
| Men | -0.03 | -0.04 | 0.12 | -0.08 | -0.06 | -0.11 | -0.10 | 0.02 | -0.01 | -0.12 | -0.12 |
| n-3 PUFA, %TE |  |  |  |  |  |  |  |  |  |  |  |
| Women | 0.09 | 0.07 | 0.09 | 0.04 | 0.06 | -0.03 | -0.02 | 0.03 | 0.12 | 0.01 | -0.02 |
| Men | 0.09 | 0.09 | 0.09 | -0.06 | 0.07 | 0.003 | 0.02 | 0.11 | -0.04 | 0.05 | -0.03 |
| n-6 PUFA, %TE |  |  |  |  |  |  |  |  |  |  |  |
| Women | -0.04 | -0.05 | 0.06 | -0.02 | -0.06 | -0.09 | -0.08 | -0.17* | 0.01 | -0.21** | 0.10 |
| Men | 0.03 | 0.03 | 0.12 | -0.06 | -0.01 | -0.09 | -0.06 | 0.01 | 0.04 | -0.25* | -0.11 |
| Trans-fat, %TE |  |  |  |  |  |  |  |  |  |  |  |
| Women | 0.18* | 0.19** | 0.01 | 0.03 | 0.20** | 0.14* | 0.15* | 0.10 | 0.10 | -0.02 | 0.07 |
| Men | 0.23** | 0.21** | 0.11 | 0.02 | 0.22** | 0.10 | 0.11 | 0.13 | 0.07 | -0.01 | 0.10 |
| Protein, %TE |  |  |  |  |  |  |  |  |  |  |  |
| Women | 0.03 | 0.03 | 0.04 | 0.01 | 0.01 | -0.01 | 0.01 | 0.11 | 0.06 | 0.06 | 0.10 |
| Men | 0.13 | 0.13 | 0.09 | -0.03 | 0.11 | 0.03 | 0.04 | -0.06 | 0.02 | 0.05 | 0.05 |
| Carbohydrate, %TE |  |  |  |  |  |  |  |  |  |  |  |
| Women | -0.19** | -0.17* | -0.14* | -0.06 | -0.16* | -0.02 | -0.04 | -0.06 | -0.17* | -0.05 | -0.14* |
| Men | -0.24** | -0.21** | -0.23** | 0.08 | -0.19** | -0.01 | -0.04 | -0.05 | -0.08 | 0.06 | 0.01 |

^1^Data analysed by Spearman’s correlations

*Significant differences at the 0.05 level

**Significant differences at the 0.01 level

^2^Abbreviations: CRP: C-reactive protein; HDL-C: high-density lipoprotein-cholesterol; LDL-C: low-density lipoprotein-cholesterol; MUFA: monounsaturated fatty acids; NEFA: non-esterified fatty acids; PUFA: polyunsaturated fatty acids; SFA: saturated fatty acids; TC: total cholesterol; TAG: triacylglycerol; % TE: % of total energy.

**Supplemental Table 3** Spearman’s correlation coefficients (r_s_) for the relationship between DXA body composition measurements, CVD risk factors and dietary macronutrients in men women ^1,2^

|  | Body fat, % | Fat mass, kg | Lean mass, kg | VAT, g | Android fat, kg | Android fat % | | Gynoid fat % | A/G |
| --- | --- | --- | --- | --- | --- | --- | --- | --- | --- |
| **Blood pressure, mmHg** | |  |  |  |  |  | |  |  |
| Systolic |  |  |  |  |  |  | |  |  |
| Women | 0.25** | 0.29** | 0.18* | 0.43** | 0.31** | 0.27** | | 0.18* | 0.25** |
| Men | 0.06 | 0.08 | 0.08 | 0.13 | 0.11 | 0.09 | | -0.01 | 0.19* |
| Diastolic |  |  |  |  |  |  | |  |  |
| Women | 0.30** | 0.31** | 0.15* | 0.37** | 0.32** | 0.31** | | 0.24** | 0.28** |
| Men | 0.33** | 0.32** | -0.04 | 0.41** | 0.37** | 0.37** | | 0.25** | 0.40** |
| Pulse pressure |  |  |  |  |  |  | |  |  |
| Women | 0.07 | 0.12 | 0.14* | 0.25** | 0.13 | 0.08 | | 0.02 | 0.09 |
| Men | -0.15 | -0.11 | 0.14 | -0.14 | -0.13 | -0.15 | | -0.16* | -0.08 |
| **Biochemistry** |  |  |  |  |  |  | |  |  |
| TC, mmol/L |  |  |  |  |  |  | |  |  |
| Women | 0.22** | 0.16* | -0.12 | 0.33** | 0.22** | 0.22** | | 0.15* | 0.22** |
| Men | 0.21** | 0.18* | -0.08 | 0.31** | 0.26** | 0.26** | | 0.15 | 0.31** |
| TAG, mmol/L |  |  |  |  |  |  | |  |  |
| Women | 0.24** | 0.25** | 0.09 | 0.34** | 0.24** | 0.27** | | 0.15* | 0.29** |
| Men | 0.49** | 0.48** | -0.01 | 0.48** | 0.53** | 0.53** | | 0.43** | 0.46** |
| HDL-C, mmol/L |  |  |  |  |  |  | |  |  |
| Women | -0.15* | -0.17* | -0.10 | -0.04 | -0.13 | -0.15* | | -0.13 | -0.13 |
| Men | -0.37** | -0.39** | -0.04 | -0.30** | -0.35** | -0.34** | | -0.37** | -0.19* |
| LDL-C, mmol/L |  |  |  |  |  |  | |  |  |
| Women | 0.25** | 0.18* | -0.12 | 0.31** | 0.24** | 0.24** | | 0.20** | 0.21** |
| Men | 0.25** | 0.23** | -0.09 | 0.34** | 0.29** | 0.28** | | 0.19* | 0.31** |
| Non-HDL-C, mmol/L |  |  |  |  |  |  | |  |  |
| Women | 0.30** | 0.23** | -0.10 | 0.37** | 0.29** | 0.30** | | 0.23** | 0.28** |
| Men | 0.34** | 0.32** | -0.07 | 0.43** | 0.38** | 0.38** | | 0.26** | 0.39** |
| TC: HDL ratio |  |  |  |  |  |  | |  |  |
| Women | 0.34** | 0.29** | -0.03 | 0.33** | 0.31** | 0.33** | | 0.27** | 0.31** |
| Men | 0.52** | 0.51** | -0.05 | 0.54** | 0.54** | 0.54** | | 0.45** | 0.46** |
| LDL-C: HDL-C ratio |  |  |  |  |  |  | |  |  |
| Women | 0.31** | 0.26** | -0.03 | 0.30** | 0.28** | 0.29** | | 0.26** | 0.25** |
| Men | 0.47** | 0.45** | -0.08 | 0.49** | 0.49** | 0.48** | | 0.41** | 0.41** |
| NEFA, μmol/L |  |  |  |  |  |  | |  |  |
| Women | 0.14 | 0.08 | -0.11 | 0.13 | 0.11 | 0.12 | | 0.14 | 0.08 |
| Men | 0.19* | 0.14 | -0.16* | 0.10 | 0.14 | 0.16* | | 0.21** | 0.04 |
| Glucose, mmol/L |  |  |  |  |  |  | |  |  |
| Women | 0.24** | 0.26** | 0.14 | 0.36** | 0.28** | 0.26** | | 0.26** | 0.22** |
| Men | 0.22** | 0.23** | 0.08 | 0.35** | 0.28** | 0.26** | | 0.19* | 0.30** |
| Insulin, pmol/L |  |  |  |  |  |  | |  |  |
| Women | 0.36** | 0.32** | -0.02 | 0.28** | 0.32** | 0.34** | | 0.31** | 0.31** |
| Men | 0.51** | 0.52** | 0.04 | 0.51** | 0.53** | 0.53** | | 0.42** | 0.49** |
| HOMA-IR |  |  |  |  |  |  | |  |  |
| Women | 0.35** | 0.32** | 0.00 | 0.31** | 0.33** | 0.33** | | 0.30** | 0.31** |
| Men | 0.51** | 0.53** | 0.08 | 0.55** | 0.55** | 0.54** | | 0.43** | 0.50** |
| CRP, mg/L |  |  |  |  |  |  | |  |  |
| Women | 0.37** | 0.37** | -0.001 | 0.27** | 0.33** | 0.36** | | 0.32** | 0.31** |
| Men | 0.45** | 0.46** | 0.07 | 0.43** | 0.48** | 0.47** | | 0.42** | 0.37** |
| GGT, U/L |  |  |  |  |  |  | |  |  |
| Women | 0.05 | 0.10 | 0.16* | 0.17* | 0.11 | 0.08 | | -0.02 | 0.13 |
| Men | 0.29** | 0.30** | 0.09 | 0.29** | 0.33** | 0.32** | | 0.29** | 0.27** |
| Uric acid, µmol/L |  |  |  |  |  |  | |  |  |
| Women | 0.20** | 0.23** | 0.09 | 0.13 | 0.25** | 0.23** | | 0.15* | 0.25** |
| Men | 0.22** | 0.23** | 0.03 | 0.16* | 0.22** | 0.24** | | 0.20** | 0.18* |
| Adiponectin, µg/mL |  |  |  |  |  |  | |  |  |
| Women | 0.05 | 0.001 | -0.07 | -0.01 | 0.01 | 0.01 | | 0.09 | -0.06 |
| Men | 0.02 | 0.004 | -0.07 | 0.01 | -0.01 | -0.02 | | 0.06 | -0.14 |
| Total 25(OH)D, ng/mL |  |  |  |  |  |  | |  |  |
| Women | -0.19** | -0.10 | 0.19** | -0.02 | -0.07 | -0.14 | | -0.25** | -0.04 |
| Men | -0.18* | -0.17* | 0.06 | -0.11 | -0.14 | -0.16* | | -0.19* | -0.05 |
| **Dietary Intake** |  |  |  |  |  |  | |  |  |
| Total fat, %TE |  |  |  |  |  |  | |  |  |
| Women | 0.02 | 0.03 | 0.04 | 0.11 | 0.05 | 0.06 | | -0.03 | 0.11 |
| Men | -0.04 | -0.02 | 0.06 | 0.02 | -0.01 | -0.04 | | -0.05 | -0.02 |
| SFA %TE |  |  |  |  |  |  | |  |  |
| Women | 0.04 | 0.05 | 0.07 | 0.11 | 0.03 | 0.03 | | 0.004 | 0.04 |
| Men | 0.05 | 0.08 | 0.06 | 0.12 | 0.09 | 0.06 | | 0.04 | 0.08 |
| MUFA, %TE |  |  |  |  |  |  | |  |  |
| Women | 0.01 | 0.03 | 0.03 | 0.08 | 0.05 | 0.05 | | -0.03 | 0.10 |
| Men | -0.07 | -0.04 | 0.04 | -0.01 | -0.03 | -0.06 | | -0.08 | -0.01 |
| PUFA, %TE |  |  |  |  |  |  | |  |  |
| Women | -0.12 | -0.12 | -0.04 | -0.01 | -0.09 | -0.07 | | -0.17* | 0.01 |
| Men | -0.17* | -0.18* | -0.02 | -0.15* | -0.18* | -0.18* | | -0.15 | -0.18* |
| n-6 PUFA, %TE |  |  |  |  |  |  | |  |  |
| Women | -0.15* | -0.12 | 0.08 | -0.06 | -0.13 | -0.12 | | -0.20** | -0.03 |
| Men | -0.22** | -0.24** | -0.08 | -0.20** | -0.23** | -0.22** | | -0.17* | -0.22** |
| n-3 PUFA, %TE |  |  |  |  |  | |  |  |  |
| Women | 0.05 | 0.004 | -0.13 | 0.12 | 0.04 | | 0.09 | 0.04 | 0.10 |
| Men | -0.10 | -0.11 | 0.003 | -0.02 | -0.10 | | -0.12 | -0.09 | -0.09 |
| Trans fat, %TE |  |  |  |  |  | |  |  |  |
| Women | 0.12 | 0.10 | -0.001 | 0.15* | 0.11 | | 0.13 | 0.12 | 0.11 |
| Men | 0.13 | 0.15* | 0.04 | 0.17* | 0.16* | | 0.14 | 0.12 | 0.11 |
| Protein, %TE |  |  |  |  |  | |  |  |  |
| Women | 0.00 | 0.04 | 0.16* | 0.02 | 0.03 | | -0.01 | -0.02 | -0.01 |
| Men | -0.09 | -0.04 | 0.16* | -0.09 | -0.06 | | -0.09 | -0.10 | -0.08 |
| Carbohydrate, %TE |  |  |  |  |  | |  |  |  |
| Women | 0.01 | -0.05 | -0.17* | -0.14* | -0.05 | | -0.03 | 0.06 | -0.07 |
| Men | 0.09 | 0.04 | -0.15 | -0.01 | 0.03 | | 0.07 | 0.10 | 0.04 |
| Fibre (AOAC), g/day |  |  |  |  |  | |  |  |  |
| Women | -0.26** | -0.16* | 0.26** | -0.13 | -0.15* | | -0.25** | -0.26** | -0.20** |
| Men | -0.13 | -0.10 | 0.12 | -0.13 | -0.12 | | -0.14 | -0.11 | -0.12 |
| Total Sugars, %TE |  |  |  |  |  | |  |  |  |
| Women | -0.09 | -0.06 | 0.05 | -0.12 | -0.04 | | -0.09 | -0.12 | -0.05 |
| Men | 0.03 | 0.01 | -0.04 | -0.01 | 0.003 | | -0.01 | 0.05 | -0.02 |

^1^Data analysed by Spearman’s correlations

*Significant differences at the 0.05 level

**Significant differences at the 0.01 level

^2^Abbreviations: AOAC: association of analytical chemists; A/G: android to gynoid ratio; CRP: C-reactive protein; GGT: gamma-glutamyl transferase; HDL-C: high density lipoprotein cholesterol; HOMA-IR: homeostatic model assessment for insulin resistance; LDL-C: low density lipoprotein cholesterol; MUFA: monounsaturated fatty acids; NEFA: non-esterified fatty acids; PUFA: polyunsaturated fatty acids; SFA: saturated fatty acids; TC: total cholesterol; TAG: triacylglycerol; %TE: % of total energy; VAT: abdominal visceral adipose tissue; total 25(OH)D: 25-hydroxyvitamin D.
